# Ethyl 1-benzene­sulfonyl-2-[(*E*)-2-(2-methyl­phen­yl)ethen­yl]indole-3-carboxyl­ate

**DOI:** 10.1107/S1600536811001863

**Published:** 2011-01-22

**Authors:** C. Ramathilagam, V. Saravanan, A. K. Mohanakrishnan, G. Chakkaravarthi, P. R. Umarani, V. Manivannan

**Affiliations:** a Department of Physics, AMET University, Kanathur, Chennai 603 112, India; b Department of Organic Chemistry, University of Madras, Guindy Campus, Chennai 600 025, India; c Department of Physics, CPCL Polytechnic College, Chennai 600 068, India; d Department of Physics, Presidency College (Autonomous), Chennai 600 005, India; e Department of Research and Development, PRIST University, Vallam, Thanjavur 613 403, Tamil Nadu, India

## Abstract

In the title compound, C_26_H_23_NO_4_S, the phenyl, tolyl and ester groups make dihedral angles of 82.28 (5), 77.67 (6) and 8.52 (6)°, respectively, with the indole ring system. The S atom of the sulfonyl group is displaced by 0.1968 (4) Å from the indole mean plane. The mol­ecular structure is stabilized by weak intra­molecular C—H⋯O inter­actions. The crystal structure structure features short intramolecular C—H⋯O contacts and π–π stacking inter­actions between the phenyl and tolyl groups [centroid–centroid distance = 3.9448 (11) Å].

## Related literature

For the biological activity of indole derivatives, see: Andreani *et al.* (2001 ▶); Kolocouris *et al.* (1994 ▶); Merck (1973 ▶). For the structures of closely related compounds, see: Chakkaravarthi *et al.* (2007 ▶, 2008 ▶).
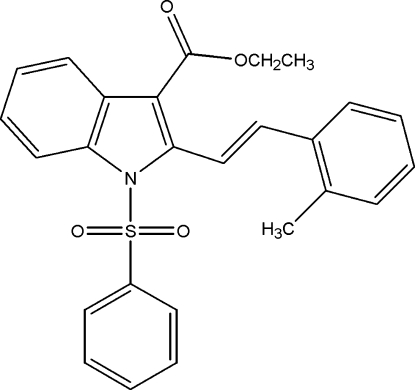


## Experimental

### 

#### Crystal data

C_26_H_23_NO_4_S
*M* *_r_* = 445.51
Monoclinic, 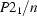

*a* = 10.4248 (4) Å
*b* = 8.3629 (3) Å
*c* = 25.2284 (11) Å
β = 92.902 (1)°
*V* = 2196.63 (15) Å^3^
*Z* = 4
Mo *K*α radiation
μ = 0.18 mm^−1^
*T* = 295 K
0.24 × 0.20 × 0.18 mm

#### Data collection

Bruker Kappa APEXII diffractometer
Absorption correction: multi-scan (*SADABS*; Sheldrick, 1996 ▶) *T*_min_ = 0.958, *T*_max_ = 0.968
22952 measured reflections
4848 independent reflections
3551 reflections with *I* > 2σ(*I*)
*R*_int_ = 0.029

#### Refinement

*R*[*F* ^2^ > 2σ(*F* ^2^)] = 0.042
*wR*(*F* ^2^) = 0.117
*S* = 1.03
4848 reflections
291 parameters
H-atom parameters constrained
Δρ_max_ = 0.25 e Å^−3^
Δρ_min_ = −0.28 e Å^−3^

### 

Data collection: *APEX2* (Bruker, 2004 ▶); cell refinement: *SAINT* (Bruker, 2004 ▶); data reduction: *SAINT*; program(s) used to solve structure: *SHELXS97* (Sheldrick, 2008 ▶); program(s) used to refine structure: *SHELXL97* (Sheldrick, 2008 ▶); molecular graphics: *PLATON* (Spek, 2009 ▶); software used to prepare material for publication: *SHELXL97*.

## Supplementary Material

Crystal structure: contains datablocks I, global. DOI: 10.1107/S1600536811001863/gk2338sup1.cif

Structure factors: contains datablocks I. DOI: 10.1107/S1600536811001863/gk2338Isup2.hkl

Additional supplementary materials:  crystallographic information; 3D view; checkCIF report

## Figures and Tables

**Table 1:** Hydrogen-bond geometry (Å, °)

*D*—H⋯*A*	*D*—H	H⋯*A*	*D*⋯*A*	*D*—H⋯*A*
C10—H10⋯O3	0.93	2.48	3.000 (3)	116
C13—H13⋯O1	0.93	2.33	2.908 (3)	120
C16—H16*A*⋯O1^i^	0.97	2.56	3.381 (3)	143

Symmetry code: (i) 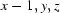.

**Fig. 1.**
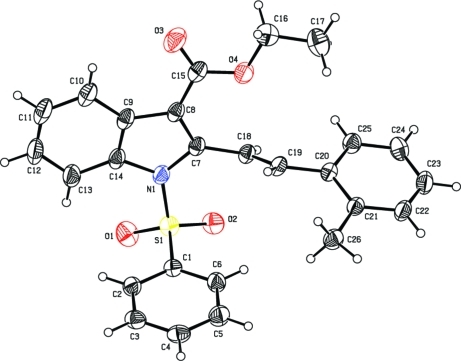
The molecular structure of the title compound with atom labels and 30% probability displacement ellipsoids for non-H atoms.

**Fig. 2.**
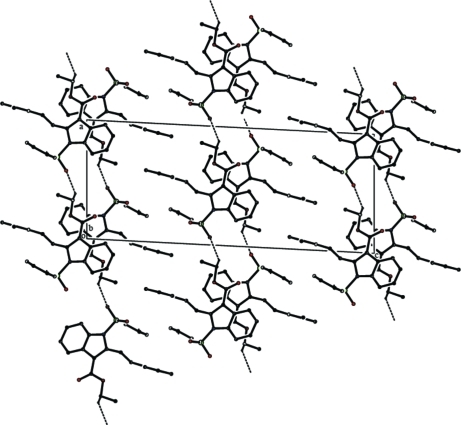
The crystal packing viewed down the b axis. Intermolecular C-H···O interactionshydrogen bonds are shown as dashed lines. H atoms not involved in hydrogen bonding have been omitted.

## supplementary crystallographic information

### Comment

Indole derivatives exhibit antihypertensive (Merck, 1973), antitumour (Andreani *et al.*, 2001) and antiviral (Kolocouris *et al.*, 1994) activities. The geometric parameters of the title molecule (Fig. 1) agree well with the reported similar structures (Chakkaravarthi *et al.* 2007, 2008).

The phenyl ring makes the dihedral angle of 82.28 (5)° with the indole ring system. The benzene ring (C20—C25) forms the dihedral angle of 77.67 (6)° with the indole ring system. The S atom of the sulfonyl group is displaced 0.1968 (4)Å from the indole mean plane. The sum of the bond angles around N1 [358.5 (1)°] indicates that N1 atom is *sp*^2^ hybridized. The molecular structure is stabilized by weak intramolecular C—H···O interactions and the crystal packing exhibits weak intermolecular C—H···O (Fig.2 and Table 1) and π–π interactions [*Cg*2···*Cg*4 (1/2 - *x*,-1/2 + *y*,1/2 - *z*) distance of 3.9448 (11) Å; *Cg*2 and *Cg*4 are the centroids of C1—C6 ring and C20—C26 ring, respectively] .

### Experimental

To a suspension of hexane (5 ml) washed NaH (0.29 g, 6.10 mmol) in dry THF (10 ml) at -10° C under N_2_ atmosphere was slowly added the solution of diethyl (3-(ethoxycarbonyl)-1-phenylsulfonyl-1*H*-indol-2-yl)methylphosphonate (0.97 g, 2.03 mmol) in dry THF (5 ml) *via* syringe and stirred for 15 min. Then a solution of 2-methylbenzaldehyde (0.28 g, 2.32 mmol) in dry THF (5 ml) was added and allowed to stir for additional 2 h. After completion of the product formation (monitored by TLC), it was then poured over crushed ice (100 g) containing conc. HCl (3 ml). The solid formed was filtered and recrystalized with MeOH to afford ethyl 2-(2-methylstyryl)-1-phenylsulfonyl -1*H*-indole-3-carboxylate as bright yellow crystals [0.70 g, 78%; melting point 371–373 K].

### Refinement

H atoms were positioned geometrically and refined using riding model approximation with C—H = 0.93 Å and *U*_iso_(H) = 1.2U_eq_(C) for aromatic C—H and C—H = 0.97 Å and *U*_iso_(H) = 1.2U_eq_(C) for methylene group and C—H = 0.96 Å and *U*_iso_(H) = 1.5U_eq_(C) for methyl group.

### Crystal data

**Table:**

C_26_H_23_NO_4_S	*F*(000) = 936
*M**_r_* = 445.51	*D*_x_ = 1.347 Mg m^−^^3^
Monoclinic, *P*2_1_/*n*	Mo *K*α radiation, λ = 0.71073 Å
Hall symbol: -P 2yn	Cell parameters from 31039 reflections
*a* = 10.4248 (4) Å	θ = 2.1–31.4°
*b* = 8.3629 (3) Å	µ = 0.18 mm^−^^1^
*c* = 25.2284 (11) Å	*T* = 295 K
β = 92.902 (1)°	Block, yellow
*V* = 2196.63 (15) Å^3^	0.24 × 0.20 × 0.18 mm
*Z* = 4	

### Data collection

**Table:**

Bruker Kappa APEXII diffractometer	4848 independent reflections
Radiation source: fine-focus sealed tube	3551 reflections with *I* > 2σ(*I*)
graphite	*R*_int_ = 0.029
ω and φ scans	θ_max_ = 27.1°, θ_min_ = 2.1°
Absorption correction: multi-scan (*SADABS*; Sheldrick, 1996)	*h* = −12→13
*T*_min_ = 0.958, *T*_max_ = 0.968	*k* = −10→10
22952 measured reflections	*l* = −32→32

### Refinement

**Table:**

Refinement on *F*^2^	Primary atom site location: structure-invariant direct methods
Least-squares matrix: full	Secondary atom site location: difference Fourier map
*R*[*F*^2^ > 2σ(*F*^2^)] = 0.042	Hydrogen site location: inferred from neighbouring sites
*wR*(*F*^2^) = 0.117	H-atom parameters constrained
*S* = 1.03	*w* = 1/[σ^2^(*F*_o_^2^) + (0.0499*P*)^2^ + 0.6907*P*] where *P* = (*F*_o_^2^ + 2*F*_c_^2^)/3
4848 reflections	(Δ/σ)_max_ < 0.001
291 parameters	Δρ_max_ = 0.25 e Å^−^^3^
0 restraints	Δρ_min_ = −0.28 e Å^−^^3^

### Fractional atomic coordinates and isotropic or equivalent isotropic displacement parameters (Å^2^)

**Table:**

	*x*	*y*	*z*	*U*_iso_*/*U*_eq_	
C1	0.29596 (16)	0.5477 (2)	0.13482 (7)	0.0434 (4)	
C2	0.33149 (19)	0.4055 (2)	0.11194 (7)	0.0516 (4)	
H2	0.3705	0.4051	0.0796	0.062*	
C3	0.3082 (2)	0.2640 (2)	0.13788 (8)	0.0563 (5)	
H3	0.3314	0.1672	0.1229	0.068*	
C4	0.2511 (2)	0.2656 (2)	0.18551 (8)	0.0582 (5)	
H4	0.2353	0.1697	0.2027	0.070*	
C5	0.2170 (2)	0.4074 (3)	0.20820 (8)	0.0641 (6)	
H5	0.1793	0.4073	0.2408	0.077*	
C6	0.23856 (19)	0.5502 (2)	0.18277 (8)	0.0549 (5)	
H6	0.2147	0.6466	0.1978	0.066*	
C7	0.07971 (18)	0.81521 (19)	0.06872 (6)	0.0439 (4)	
C8	−0.00146 (19)	0.7883 (2)	0.02544 (6)	0.0466 (4)	
C9	0.0676 (2)	0.7013 (2)	−0.01332 (7)	0.0505 (5)	
C10	0.0332 (2)	0.6438 (2)	−0.06437 (7)	0.0644 (6)	
H10	−0.0487	0.6611	−0.0796	0.077*	
C11	0.1231 (3)	0.5617 (3)	−0.09121 (9)	0.0782 (8)	
H11	0.1019	0.5246	−0.1253	0.094*	
C12	0.2444 (3)	0.5327 (3)	−0.06897 (9)	0.0803 (8)	
H12	0.3020	0.4733	−0.0879	0.096*	
C13	0.2824 (3)	0.5898 (3)	−0.01920 (8)	0.0698 (6)	
H13	0.3647	0.5719	−0.0045	0.084*	
C14	0.1920 (2)	0.6752 (2)	0.00782 (7)	0.0530 (5)	
C15	−0.1351 (2)	0.8425 (2)	0.01525 (7)	0.0520 (5)	
C16	−0.3192 (2)	0.9600 (3)	0.04901 (9)	0.0683 (6)	
H16A	−0.3749	0.8700	0.0401	0.082*	
H16B	−0.3273	1.0375	0.0204	0.082*	
C17	−0.3556 (3)	1.0338 (3)	0.09964 (11)	0.0900 (8)	
H17A	−0.2996	1.1223	0.1081	0.135*	
H17B	−0.3479	0.9558	0.1275	0.135*	
H17C	−0.4427	1.0710	0.0960	0.135*	
C18	0.05757 (17)	0.9015 (2)	0.11824 (6)	0.0458 (4)	
H18	0.1021	0.9965	0.1247	0.055*	
C19	−0.02112 (18)	0.8527 (2)	0.15390 (6)	0.0479 (4)	
H19	−0.0617	0.7549	0.1479	0.057*	
C20	−0.05081 (16)	0.9393 (2)	0.20259 (7)	0.0445 (4)	
C21	−0.07577 (16)	0.8570 (2)	0.24966 (6)	0.0460 (4)	
C22	−0.10097 (19)	0.9451 (3)	0.29430 (7)	0.0570 (5)	
H22	−0.1176	0.8917	0.3255	0.068*	
C23	−0.1022 (2)	1.1089 (3)	0.29386 (8)	0.0644 (6)	
H23	−0.1181	1.1652	0.3246	0.077*	
C24	−0.0800 (2)	1.1895 (3)	0.24804 (9)	0.0644 (6)	
H24	−0.0816	1.3007	0.2475	0.077*	
C25	−0.05529 (19)	1.1053 (2)	0.20262 (8)	0.0543 (5)	
H25	−0.0414	1.1607	0.1715	0.065*	
C26	−0.0706 (2)	0.6777 (2)	0.25305 (8)	0.0594 (5)	
H26A	−0.0929	0.6442	0.2878	0.089*	
H26B	0.0146	0.6417	0.2465	0.089*	
H26C	−0.1304	0.6326	0.2270	0.089*	
N1	0.20131 (15)	0.75147 (17)	0.05839 (6)	0.0494 (4)	
O1	0.43527 (14)	0.70935 (19)	0.07195 (6)	0.0717 (4)	
O2	0.31983 (13)	0.85612 (15)	0.13962 (5)	0.0603 (4)	
O3	−0.19140 (16)	0.8300 (2)	−0.02733 (6)	0.0866 (5)	
O4	−0.18768 (13)	0.90745 (18)	0.05650 (5)	0.0618 (4)	
S1	0.32570 (5)	0.72867 (5)	0.10266 (2)	0.05081 (15)	

### Atomic displacement parameters (Å^2^)

**Table:**

	*U*^11^	*U*^22^	*U*^33^	*U*^12^	*U*^13^	*U*^23^
C1	0.0433 (9)	0.0401 (8)	0.0464 (9)	−0.0039 (7)	−0.0009 (7)	0.0010 (7)
C2	0.0587 (12)	0.0467 (10)	0.0492 (10)	0.0004 (8)	0.0014 (9)	−0.0044 (8)
C3	0.0654 (13)	0.0397 (9)	0.0624 (12)	0.0005 (9)	−0.0099 (10)	−0.0035 (8)
C4	0.0644 (13)	0.0464 (10)	0.0625 (12)	−0.0059 (9)	−0.0087 (10)	0.0116 (9)
C5	0.0771 (15)	0.0591 (12)	0.0571 (12)	−0.0003 (11)	0.0132 (10)	0.0104 (10)
C6	0.0661 (12)	0.0454 (10)	0.0538 (11)	0.0034 (9)	0.0104 (9)	0.0007 (8)
C7	0.0591 (11)	0.0362 (8)	0.0371 (8)	−0.0067 (8)	0.0108 (8)	0.0032 (7)
C8	0.0653 (12)	0.0390 (9)	0.0363 (9)	−0.0110 (8)	0.0094 (8)	0.0019 (7)
C9	0.0780 (14)	0.0366 (9)	0.0380 (9)	−0.0125 (9)	0.0134 (9)	0.0027 (7)
C10	0.1043 (17)	0.0483 (11)	0.0412 (10)	−0.0157 (11)	0.0112 (10)	−0.0031 (9)
C11	0.146 (3)	0.0495 (12)	0.0414 (11)	−0.0110 (14)	0.0231 (14)	−0.0058 (9)
C12	0.139 (2)	0.0523 (12)	0.0534 (13)	0.0117 (14)	0.0431 (15)	0.0011 (10)
C13	0.0991 (17)	0.0592 (12)	0.0537 (12)	0.0121 (12)	0.0293 (12)	0.0066 (10)
C14	0.0827 (14)	0.0383 (9)	0.0396 (9)	−0.0030 (9)	0.0199 (9)	0.0045 (7)
C15	0.0654 (12)	0.0482 (10)	0.0426 (10)	−0.0170 (9)	0.0037 (9)	0.0007 (8)
C16	0.0565 (13)	0.0702 (14)	0.0784 (15)	−0.0075 (11)	0.0065 (11)	0.0081 (12)
C17	0.0768 (17)	0.0912 (18)	0.104 (2)	0.0034 (14)	0.0249 (15)	−0.0120 (16)
C18	0.0572 (11)	0.0414 (9)	0.0389 (9)	−0.0050 (8)	0.0039 (8)	−0.0034 (7)
C19	0.0635 (12)	0.0429 (9)	0.0376 (9)	−0.0074 (8)	0.0051 (8)	−0.0013 (7)
C20	0.0440 (10)	0.0504 (10)	0.0390 (9)	−0.0034 (8)	0.0024 (7)	−0.0036 (7)
C21	0.0398 (9)	0.0587 (11)	0.0394 (9)	−0.0019 (8)	0.0019 (7)	−0.0001 (8)
C22	0.0562 (12)	0.0771 (14)	0.0380 (9)	0.0023 (10)	0.0074 (8)	−0.0026 (9)
C23	0.0654 (13)	0.0780 (15)	0.0504 (11)	0.0045 (11)	0.0079 (10)	−0.0189 (11)
C24	0.0672 (14)	0.0521 (11)	0.0745 (14)	0.0019 (10)	0.0106 (11)	−0.0151 (10)
C25	0.0615 (12)	0.0514 (10)	0.0507 (10)	−0.0026 (9)	0.0085 (9)	0.0002 (9)
C26	0.0654 (13)	0.0607 (12)	0.0528 (11)	−0.0022 (10)	0.0115 (10)	0.0078 (9)
N1	0.0647 (10)	0.0456 (8)	0.0386 (8)	0.0016 (7)	0.0102 (7)	0.0027 (6)
O1	0.0589 (9)	0.0704 (10)	0.0881 (11)	−0.0103 (7)	0.0267 (8)	0.0072 (8)
O2	0.0694 (9)	0.0408 (7)	0.0699 (9)	−0.0097 (6)	−0.0036 (7)	−0.0054 (6)
O3	0.0824 (11)	0.1226 (15)	0.0535 (9)	−0.0018 (10)	−0.0107 (8)	−0.0151 (9)
O4	0.0587 (9)	0.0764 (9)	0.0504 (8)	0.0003 (7)	0.0037 (6)	−0.0047 (7)
S1	0.0530 (3)	0.0423 (2)	0.0579 (3)	−0.0081 (2)	0.0101 (2)	0.0021 (2)

### Geometric parameters (Å, °)

**Table:**

C1—C6	1.377 (3)	C15—O4	1.318 (2)
C1—C2	1.381 (2)	C16—O4	1.444 (2)
C1—S1	1.7523 (17)	C16—C17	1.485 (3)
C2—C3	1.379 (3)	C16—H16A	0.9700
C2—H2	0.9300	C16—H16B	0.9700
C3—C4	1.368 (3)	C17—H17A	0.9600
C3—H3	0.9300	C17—H17B	0.9600
C4—C5	1.371 (3)	C17—H17C	0.9600
C4—H4	0.9300	C18—C19	1.313 (2)
C5—C6	1.379 (3)	C18—H18	0.9300
C5—H5	0.9300	C19—C20	1.472 (2)
C6—H6	0.9300	C19—H19	0.9300
C7—C8	1.366 (2)	C20—C25	1.389 (3)
C7—N1	1.412 (2)	C20—C21	1.408 (2)
C7—C18	1.471 (2)	C21—C22	1.382 (3)
C8—C9	1.440 (2)	C21—C26	1.503 (3)
C8—C15	1.475 (3)	C22—C23	1.370 (3)
C9—C14	1.394 (3)	C22—H22	0.9300
C9—C10	1.404 (3)	C23—C24	1.368 (3)
C10—C11	1.368 (3)	C23—H23	0.9300
C10—H10	0.9300	C24—C25	1.380 (3)
C11—C12	1.379 (4)	C24—H24	0.9300
C11—H11	0.9300	C25—H25	0.9300
C12—C13	1.383 (3)	C26—H26A	0.9600
C12—H12	0.9300	C26—H26B	0.9600
C13—C14	1.388 (3)	C26—H26C	0.9600
C13—H13	0.9300	N1—S1	1.6789 (17)
C14—N1	1.425 (2)	O1—S1	1.4217 (14)
C15—O3	1.202 (2)	O2—S1	1.4194 (14)
			
C6—C1—C2	121.21 (17)	C17—C16—H16B	110.3
C6—C1—S1	119.25 (13)	H16A—C16—H16B	108.5
C2—C1—S1	119.54 (14)	C16—C17—H17A	109.5
C3—C2—C1	118.89 (18)	C16—C17—H17B	109.5
C3—C2—H2	120.6	H17A—C17—H17B	109.5
C1—C2—H2	120.6	C16—C17—H17C	109.5
C4—C3—C2	120.21 (18)	H17A—C17—H17C	109.5
C4—C3—H3	119.9	H17B—C17—H17C	109.5
C2—C3—H3	119.9	C19—C18—C7	124.04 (16)
C3—C4—C5	120.57 (18)	C19—C18—H18	118.0
C3—C4—H4	119.7	C7—C18—H18	118.0
C5—C4—H4	119.7	C18—C19—C20	125.73 (17)
C4—C5—C6	120.19 (19)	C18—C19—H19	117.1
C4—C5—H5	119.9	C20—C19—H19	117.1
C6—C5—H5	119.9	C25—C20—C21	118.73 (16)
C1—C6—C5	118.93 (18)	C25—C20—C19	120.03 (16)
C1—C6—H6	120.5	C21—C20—C19	121.24 (16)
C5—C6—H6	120.5	C22—C21—C20	118.50 (18)
C8—C7—N1	108.42 (15)	C22—C21—C26	119.57 (17)
C8—C7—C18	129.95 (17)	C20—C21—C26	121.89 (16)
N1—C7—C18	121.54 (16)	C23—C22—C21	121.92 (19)
C7—C8—C9	108.39 (18)	C23—C22—H22	119.0
C7—C8—C15	129.07 (16)	C21—C22—H22	119.0
C9—C8—C15	122.45 (17)	C24—C23—C22	119.83 (19)
C14—C9—C10	119.08 (19)	C24—C23—H23	120.1
C14—C9—C8	107.85 (16)	C22—C23—H23	120.1
C10—C9—C8	133.1 (2)	C23—C24—C25	119.8 (2)
C11—C10—C9	118.5 (2)	C23—C24—H24	120.1
C11—C10—H10	120.8	C25—C24—H24	120.1
C9—C10—H10	120.8	C24—C25—C20	121.19 (19)
C10—C11—C12	121.6 (2)	C24—C25—H25	119.4
C10—C11—H11	119.2	C20—C25—H25	119.4
C12—C11—H11	119.2	C21—C26—H26A	109.5
C11—C12—C13	121.6 (2)	C21—C26—H26B	109.5
C11—C12—H12	119.2	H26A—C26—H26B	109.5
C13—C12—H12	119.2	C21—C26—H26C	109.5
C12—C13—C14	116.9 (2)	H26A—C26—H26C	109.5
C12—C13—H13	121.5	H26B—C26—H26C	109.5
C14—C13—H13	121.5	C7—N1—C14	108.25 (16)
C13—C14—C9	122.30 (19)	C7—N1—S1	126.20 (12)
C13—C14—N1	130.7 (2)	C14—N1—S1	124.06 (14)
C9—C14—N1	106.99 (16)	C15—O4—C16	116.92 (16)
O3—C15—O4	122.5 (2)	O2—S1—O1	120.39 (9)
O3—C15—C8	122.88 (19)	O2—S1—N1	107.11 (8)
O4—C15—C8	114.58 (16)	O1—S1—N1	105.37 (9)
O4—C16—C17	107.22 (19)	O2—S1—C1	109.27 (8)
O4—C16—H16A	110.3	O1—S1—C1	108.67 (9)
C17—C16—H16A	110.3	N1—S1—C1	104.91 (8)
O4—C16—H16B	110.3		
			
C6—C1—C2—C3	0.2 (3)	C18—C19—C20—C21	−146.01 (19)
S1—C1—C2—C3	179.60 (15)	C25—C20—C21—C22	−1.4 (3)
C1—C2—C3—C4	−0.2 (3)	C19—C20—C21—C22	178.96 (17)
C2—C3—C4—C5	−0.4 (3)	C25—C20—C21—C26	−178.99 (18)
C3—C4—C5—C6	0.8 (3)	C19—C20—C21—C26	1.4 (3)
C2—C1—C6—C5	0.3 (3)	C20—C21—C22—C23	0.0 (3)
S1—C1—C6—C5	−179.13 (16)	C26—C21—C22—C23	177.64 (19)
C4—C5—C6—C1	−0.8 (3)	C21—C22—C23—C24	1.0 (3)
N1—C7—C8—C9	1.62 (18)	C22—C23—C24—C25	−0.6 (3)
C18—C7—C8—C9	178.34 (16)	C23—C24—C25—C20	−0.8 (3)
N1—C7—C8—C15	−174.87 (16)	C21—C20—C25—C24	1.8 (3)
C18—C7—C8—C15	1.8 (3)	C19—C20—C25—C24	−178.52 (18)
C7—C8—C9—C14	0.51 (19)	C8—C7—N1—C14	−3.13 (18)
C15—C8—C9—C14	177.28 (15)	C18—C7—N1—C14	179.82 (14)
C7—C8—C9—C10	−179.41 (18)	C8—C7—N1—S1	−169.56 (12)
C15—C8—C9—C10	−2.6 (3)	C18—C7—N1—S1	13.4 (2)
C14—C9—C10—C11	1.0 (3)	C13—C14—N1—C7	−177.09 (18)
C8—C9—C10—C11	−179.08 (19)	C9—C14—N1—C7	3.40 (18)
C9—C10—C11—C12	1.0 (3)	C13—C14—N1—S1	−10.3 (3)
C10—C11—C12—C13	−2.2 (4)	C9—C14—N1—S1	170.19 (12)
C11—C12—C13—C14	1.2 (3)	O3—C15—O4—C16	1.7 (3)
C12—C13—C14—C9	0.9 (3)	C8—C15—O4—C16	−178.92 (16)
C12—C13—C14—N1	−178.54 (18)	C17—C16—O4—C15	−177.89 (18)
C10—C9—C14—C13	−2.0 (3)	C7—N1—S1—O2	−32.90 (16)
C8—C9—C14—C13	178.05 (17)	C14—N1—S1—O2	162.70 (14)
C10—C9—C14—N1	177.54 (15)	C7—N1—S1—O1	−162.21 (14)
C8—C9—C14—N1	−2.39 (18)	C14—N1—S1—O1	33.38 (16)
C7—C8—C15—O3	169.53 (19)	C7—N1—S1—C1	83.16 (15)
C9—C8—C15—O3	−6.5 (3)	C14—N1—S1—C1	−81.24 (15)
C7—C8—C15—O4	−9.8 (3)	C6—C1—S1—O2	17.63 (18)
C9—C8—C15—O4	174.12 (15)	C2—C1—S1—O2	−161.76 (15)
C8—C7—C18—C19	67.2 (3)	C6—C1—S1—O1	150.76 (16)
N1—C7—C18—C19	−116.4 (2)	C2—C1—S1—O1	−28.63 (18)
C7—C18—C19—C20	−176.79 (17)	C6—C1—S1—N1	−96.93 (16)
C18—C19—C20—C25	34.3 (3)	C2—C1—S1—N1	83.68 (16)

### Hydrogen-bond geometry (Å, °)

**Table:**

*D*—H···*A*	*D*—H	H···*A*	*D*···*A*	*D*—H···*A*
C10—H10···O3	0.93	2.48	3.000 (3)	116
C13—H13···O1	0.93	2.33	2.908 (3)	120
C16—H16A···O1^i^	0.97	2.56	3.381 (3)	143

Symmetry codes: (i) *x*−1, *y*, *z*.

## References

[bb1] Andreani, A., Granaiola, M., Leoni, A., Locatelli, A., Morigi, R., Rambaldi, M., Giorgi, G., Salvini, L. & Garaliene, V. (2001). *Anticancer Drug Des.* **16**, 167–174. 11962514

[bb2] Bruker (2004). *APEX2* and *SAINT* Bruker AXS Inc., Madison, Wisconsin, USA.

[bb3] Chakkaravarthi, G., Dhayalan, V., Mohanakrishnan, A. K. & Manivannan, V. (2008). *Acta Cryst.* E**64**, o542. 10.1107/S1600536808003024 PMC2960400 21201561

[bb4] Chakkaravarthi, G., Ramesh, N., Mohanakrishnan, A. K. & Manivannan, V. (2007). *Acta Cryst.* E**63**, o3564.

[bb5] Kolocouris, N., Foscolos, G. B., Kolocouris, A., Marakos, P., Pouli, N., Fytas, G., Ikeda, S. & De Clercq, E. (1994). *J. Med. Chem.* **37**, 2896–2902. 10.1021/jm00044a010 8071937

[bb6] Merck (1973). French Patent No. 2 163 554.

[bb7] Sheldrick, G. M. (1996). *SADABS*, University of Göttingen, Germany.

[bb8] Sheldrick, G. M. (2008). *Acta Cryst.* A**64**, 112–122. 10.1107/S0108767307043930 18156677

[bb9] Spek, A. L. (2009). *Acta Cryst.* D**65**, 148–155. 10.1107/S090744490804362X PMC2631630 19171970

